# Biodegradation of Polystyrene by *Tenebrio molitor*, *Galleria mellonella*, and *Zophobas atratus* Larvae and Comparison of Their Degradation Effects

**DOI:** 10.3390/polym13203539

**Published:** 2021-10-14

**Authors:** Shan Jiang, Tingting Su, Jingjing Zhao, Zhanyong Wang

**Affiliations:** 1 School of Petrochemical Engineering, Liaoning Petrochemical University, Fushun 113001, China; js18341318089@163.com (S.J.); sutingting1978@126.com (T.S.); 2 Department of Biotechnology, College of Bioscience and Biotechnology, Shenyang Agricultural University, Shenyang 110866, China

**Keywords:** biodegradation, polystyrene, comparison, gut microbes, insect larvae

## Abstract

Plastic waste pollution and its difficult degradation process have aroused widespread concern. Research has demonstrated that the larvae of *Tenebrio molitor* (yellow mealworm), *Galleria mellonella* (greater wax moth), and *Zophobas atratus* (superworm) possess a biodegradation ability for polystyrene (PS) within the gut microbiota of these organisms. In this study, the difference in PS degradation and the changes of the gut microbiota were compared before and after feeding PS. The results showed that superworm had the strongest PS consumption capacity and the highest survival rate during the 30 d experiment period. They all could degrade PS to different degrees. Superworm showed the highest ability to degrade PS into low-molecular-weight substances, while yellow mealworm depolymerized PS strongly by destroying the benzene ring. The changes of the intestinal microbiome caused by feeding PS showed that after ingesting PS, there was a decrease in community diversity in superworm and yellow mealworm, but an increase in greater wax moth. Meanwhile, Enterococcus and Enterobacteriaceae, found in all three species’ larvae upon 20 d of PS feeding, might play an important role in PS degradation. The results will provide more accurate PS degradation comparative data of the three species’ larvae and theoretical guidance for further research on the efficient PS biodegradations.

## 1. Introduction

It is generally believed that petroleum-based plastics, including polyethylene (PE), polystyrene (PS), polyvinyl chloride (PVC), polypropylene (PP), etc., are widely used due to their light weight, high strength, waterproof capacity, corrosion resistance, and low cost [1]. The convenience of using plastics by humans has led to the release of a large amount of plastic waste into the environment. These waste plastics have stable chemical properties, thus making it extremely difficult to naturally degrade them in the environment [2,3]. Therefore, they lead to the pollution of the soil, atmosphere, and water to different degrees. These wastes breakdown into microplastics, which might enter an organism’s body and accumulate there, seriously endangering the life and health of the organism [4].

Some of the traditional methods of disposing of waste plastic include incineration, landfill, or chemical recycling, which cannot fundamentally solve the problem of environmental pollution. Thus, biodegradation is an ideal way to solve this problem [5,6]. Previous studies have been conducted on the biodegradation of plastic, in which several bacteria and fungi were found to be capable of degrading plastic materials [7,8,9]. In recent years, more and more insect larvae have been found to possess the ability to feed on, degrade, and mineralize plastics, such as *Tenebrio molitor* L., *Galleria mellonella* L., *Zophobas atratus* Fab., *Tenebrio obscurus* Fab., *Plodia interpunctella* Htibner, *Tribolium castaneum* Herbst, *Lasioderma serricorne* F., *Rhyzopertha dominica* F., and *Sitophilus oryzae* L. [10,11,12,13,14,15,16,17,18]. Meanwhile, the plastic consumption rate of insects is higher than that of bacteria and fungi, which are isolated from various sources, such as soil, garbage, and sewage sludge [19,20,21]. Among these insect larvae, three species have been studied further, including the yellow mealworm (larvae of *Tenebrio molitor* L.), the greater wax moth (larvae of *Galleria mellonella* L.), and the superworm (larvae of *Zophobas atratus* Fab.). The literature and our studies have shown that all three insects can eat and degrade PS [16,22,23].

PS, as one of traditional petroleum-based plastics, is made from the polymerization of styrene monomers. The annual production of PS reaches approximately 33 million t, accounting for about 7% of the total global plastic production [24]. It is widely used in various industries, agriculture, medical treatment, and all aspects of daily life. However, PS waste is generated proportionally to its production, and a large amount of PS waste is produced every year [25]. We hope to find effective PS degradation organisms and provide the theoretical foundation to control plastic pollution. Therefore, in this study, the larvae of yellow mealworm, greater wax moth, and superworm were chosen and prepared to carry out the following research: (1) the three species of insect larvae were fed PS as their sole diet to determine and compare their feeding abilities and their survival rates; (2) the changes in the product properties of the larvae after feeding on PS were analyzed; (3) the changes in intestinal microbiome of the larvae after feeding on PS were compared for all three species’ larvae.

## 2. Materials and Methods

### 2.1. Test Materials

*G. mellonella* larvae (15–20 mm long) and beeswax were purchased from Huiyude Co. (Tianjin, China). The larvae of *T. molitor* (20–25 mm long) and *Z. atratus* (30–40 mm long) and wheat bran were purchased from Hongqiao Insect Breeding Plant (Tianjin, China). Prior to the tests, *G. mellonella* larvae were fed with beeswax, while the larvae of *T. molitor* and *Z. atratus* were fed with wheat bran. None of the larvae were fed with any kind of antibiotics or additives. The larvae were starved for 36 h before the experiment to avoid any effect of the previously eaten food.

Styrofoam (PS foam) was obtained from SINOPEC Beijing Yanshan Company (Beijing, China). No catalysts and additives were added as per the manufacturing standard in China (QB/T 4009-2010). The number-averaged molecular weight (*M_n_*) and weight-averaged molecular weight (*M_w_*) were measured by GPC, and the values were 64,400 Da and 144,400 Da, respectively.

### 2.2. Feeding Tests

Each species’ larvae were divided into two groups (200 larvae per group). Each group was reared in a polypropylene plastic container (L × W × H = 14 cm × 14 cm × 7 cm), and the treated group was fed with Styrofoam blocks (3.0 g a group) as the sole diet. As a control, *G. mellonella* larvae were reared on beeswax (3.0 g), and the larvae of *T. molitor* and *Z. atratus* were reared on wheat bran (3.0 g), respectively. Additional Styrofoam blocks and bran were added for 3 d to maintain adequate diet within each container. Both the test group and the control group were prepared in triplicate (n = 3). The measurement of the survival rates and plastic mass loss caused by the larval activities was carried out every 5 d and ended on Day 30. During the testing time, dead larvae and molted exoskeletons were removed from the containers immediately to prevent them from being eaten by the remaining larvae, as cannibalism existed in the later period. Containers were stored in a controlled environment maintained at 25 ± 2 °C.

### 2.3. Collection and Characterization of Frass

The biodegradation assay was characterized by its weight loss and change in bonding upon transforming the PS foam to frass. To obtain enough frass for characterization, additional larvae (approximately 1000 for each group) were fed with Styrofoam blocks as their sole diet for 21 d. After the period, larvae were transferred to a clean container for the collection of frass every 12 h. In this way, the carryover of uningested Styrofoam morsels and molted exoskeletons in the frass was avoided. The collected frass was immediately stored at −80 °C for further analysis, and the stored frass was characterized by four methods as follows, with Styrofoam foam as the control sample.

The changes in molecular weight of Styrofoam or the degradation products in frass were analyzed by gel permeation chromatography (GPC, Waters, GPC1525, Milford, MA, USA). PS extraction from PS feedstock (1.0 g) and frass samples’ (1.0 g) collection from the PS-feed larvae was performed by dissolving in tetrahydrofuran (THF) (Peng et al. 2019).

Fourier transform infrared spectroscopy (FTIR, Agilent, FTIR-660+610, Santa Clara, CA, USA) was conducted in the range from 4000–500 cm^−1^ to characterize major functional groups of PS feedstock and frass samples. Prior to the analyses, dry samples were ground with KBr to prepare a homogeneous KBr pellet for scanning.

Thermal gravimetric analysis (TGA, TA, Q600, New Castle, DE, USA) was performed to characterize thermal changes during the conversion of PS to frass. Samples of PS (5 mg) and frass (5 mg) were analyzed within a temperature range of 40–800 °C at a rate of 20 °C/min. A high-purity nitrogen flow (99.999%) was used at a rate of 20 mL/min for protection of the sample.

^1^H nuclear magnetic resonance (^1^H-NMR, Bruker BioSpin, AVANCE III HD 400, Fällanden, Switzerland) was used to characterize changes in the end group of the egested polymer at the ambient temperature. Samples were dissolved in deuterated chloroform with 99.8% purity. ^1^H-NMR spectra were measured on a 400 MHz NMR spectrometer and with ^1^H sensitivity ≥ 500:1 (0.1% EB, noise range of 200 Hz).

### 2.4. Analysis of Gut Microbial Community

Larvae of *G. mellonella*, *T. molitor*, and *Z. atratus* (200 per group) used for gut microbial community analysis were fed with PS as their sole diet and maintained under the same conditions as mentioned above. About 0.4 g of gut tissue was collected respectively on Day 0, Day 10, and Day 20 for further analysis. Prior to the dissection, larvae were immersed in 75% ethanol for 1 min and dipped into saline 3 times. Then, their guts were removed and put into a 1.5 mL centrifuge tube. The operation was performed in a sterile environment. The samples collected were stored at −80 °C until further use. The V3–V4 region of the 16S rRNA gene of the sample was sequenced using Majorbio Bio-Pharm Technology Co., Ltd. (Shanghai, China). Microbial DNA was extracted using the E.Z.N.A.^®^ Soil DNA Kit (Omega Bio-tek, Norcross, GA, USA) according to the manufacturer’s protocol. The amplicons were extracted with 2% agarose gel and further purified using the AxyPrep DNA Gel Extraction Kit (Axygen Biosciences, Union City, CA, USA). The purified amplicons were pooled in equimolar and paired-end sequenced (2 × 300) on an Illumina MiSeq platform.

### 2.5. Determination of Degradation Products

Gas chromatography–mass spectrometry (GC–MS, Agilent, Agilent 7890A/5975C, USA) was performed for further investigation of the intermediates and products generated from the biodegradation of plastic. The samples of frass and gut were collected using the process described above and pretreated based on the method mentioned by Lou et al. (2020) with modifications. The ground frass and gut from the PS-fed larvae were extracted with 10 mL of chloroform:methanol (2:1), then kept in a water bath at 55 °C for 3 h. The solvents were allowed to evaporate, and the residual polymers were redissolved in 100% hexane for the GC–MS analysis. The PS sample underwent the same pretreatment and was treated as the control group. The GC–MS used HP-5 (30 m × 250 μm × 0.25 μm) column and helium as the carrier gas. The oven temperature programming started at 40 °C followed by a hold for 4 min, an increase to 280 °C at a rate of 10 °C/min, and a 5 min hold. The compounds were identified based on the NIST17 database.

### 2.6. Statistical Analysis

Statistical ANOVAs were performed using SPSS 20.0 (SPSS Inc., Chicago, IL, USA). Pairwise comparisons were analyzed by Student’s *t*-test, as all date were normally distributed. All error values are reported as the mean value ± the standard deviation.

## 3. Results and Discussion

### 3.1. Changes in PS Consumption, Larvae Weight, and Survival Rates

**Figure 1:**
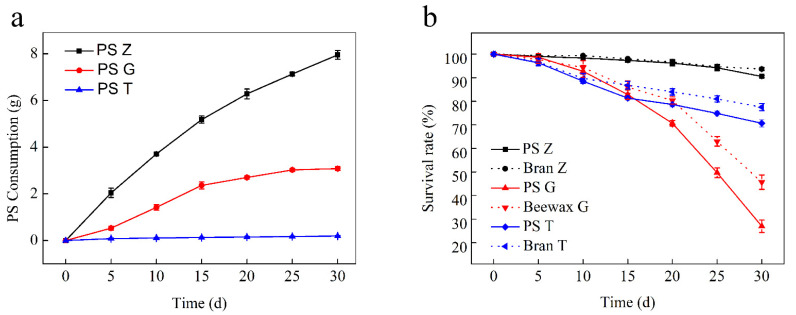
PS consumption by superworms, greater wax moths, and yellow mealworms (**a**), the survival rate for the three species’ larvae that were fed with PS and bran/beeswax (**b**). Z: superworms, G: greater wax moths, T: yellow mealworms.

When Styrofoam was placed in a container as the only diet, superworms, greater wax moths, and yellow mealworms began to feed on it and gradually produced etch loss. During the 30 d test, the PS mass consumption by superworms was 7.95 g, while that of the greater wax moths and yellow mealworms was 3.08 g and 0.19 g, respectively. There was a progressive increase in the PS consumption by all three species (Figure 1a). The average PS consumption rates were found to be 2.78 ± 0.060 mg larva^−1^ d^−1^, 1.57 ± 0.066 mg larva^−1^ d^−1^ and 0.07 ± 0.009 mg larva^−1^ d^−1^, respectively (Table 1). During the 30 d experiment period, the survival rate of larvae in the PS group and the feed group showed a downward trend, and the survival rate of the three kinds of larvae showed a significant difference. The survival rate of superworms, greater wax moths, and yellow mealworms in the feed group was 93.67 ± 1.53%, 45.67 ± 3.06%, and 80.83 ± 7.11%, respectively, and that of the PS group was 90.50 ± 0.50%, 27.00 ± 2.65%, and 75.5 ± 7.4%, respectively (Figure 1b). The results showed that the survival rate of superworms was the highest, followed by the yellow mealworm and the greater wax moths. For the same kind of larvae, the survival rate of the PS group was lower than that of the feed group.

After the experiment, the weight changes of the PS group were −51.67 ± 1.15%, −43.61 ± 4.67%, and −17.06 ± 5.28%, and the weight changes of feed group were +12.11 ± 3.37%, +18.89 ± 2.12%, and +25.92 ± 1.84%, respectively. The results showed that all three kinds of larvae were able to feed on PS; however, there were significant differences in PS consumption among the larvae of each species (Table 1). Meanwhile, a marked decrease in the mass weight of the PS-feeding larvae was observed. According to the comparison data of the survival rate and the weight changes between the PS group and feed group, it can be noted that PS cannot meet the energy needed for their growth and development. The study also showed that the PS consumption rate and weight loss of the three kinds of larvae were found to be directly proportional to their body size. The superworm was the largest in size, so the PS consumption rates were recorded as high and its body weight changed the most; yellow mealworm was the smallest, and the PS consumption rate was the lowest and its own body weight changed the least. This result is consistent with the analysis by Peng et al. [23], who stated that the greater consumption capability of superworms was likely associated with their larger size and intrinsically aggressive foraging habit.

### 3.2. Evidence and Differences of Biodegradation

**Figure 2:**
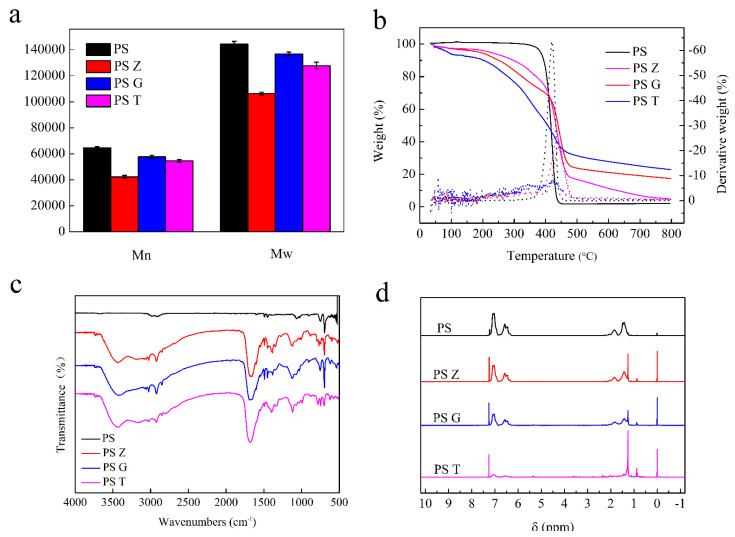
Depolymerization of PS by the three species’ larvae. Comparison of the *Mn* and *Mw* of PS and polymers extracted from frass (**a**), the TGA spectra of the PS and frass of PS-fed larvae (**b**), the FTIR spectra of the PS and frass of PS-fed larvae (**c**), and the ^1^H-NMR spectra for the PS and frass of larvae fed with PS only (**d**). Z: superworms, G: greater wax moths, T: yellow mealworms.

The THF extract of frass and the THF-dissolved pristine Styrofoam were analyzed using GPC, respectively. The results showed that the number-averaged molecular weight (*Mn*) and weight-averaged molecular weight (*Mw*) of Styrofoam were 64,400 Da and 144,400 Da, respectively. The frass extractions from superworm, greater wax moth, and yellow mealworm were 42,304 Da and 106,381 Da, 57,458 Da and 136,735 Da, 54,472 Da and 127,793 Da, respectively. It was clearly shown that the *Mn* and *Mw* of the frass of the three species’ larvae showed a decline compared to that of the Styrofoam (Figure 2a). Generally, the decline of *Mn* and *Mw* analyzed by GPC is considered as a major indication of polymer modification, depolymerization, and degradation [13,26]. These results suggested that the depolymerization of long chains of PS molecules and the lower molecular weight of the degraded products were formed in the insect larvae’s guts. Meanwhile, the decrease in both *Mn* and *Mw* of PS gave evidence of chain scission by enzymatic depolymerization or microbial attack [27,28]. In this study, the molecular weight of the degradation products of superworm was minimum, followed by yellow mealworm and greater wax moth, which indicated that the intestinal microorganisms of superworm may have the highest ability to degrade PS into low-molecular-weight substances.

TGA was used to detect the thermal modification of larvae from pristine Styrofoam to frass at the end of the 30 d test. As shown in Figure 2b, the TGA curve of Styrofoam showed only one sharp mass loss where more than 95% of the loss occurred between 380 °C and 440 °C, and the maximum decomposition rate occurred at 420 °C. In contrast, there was no significant or sudden drop detected in the TGA curves for the frass of PS-fed larvae of the three species. There was a gradual decrease in the curves from the beginning to 800 °C. A sudden drop in the PS curve represented the degradation of PS at high temperatures. However, the frass curve did not show any kind of degradation range, thus illustrating that the PS content in frass was low or absent and the biodegradation of PS took, place resulting in the formation of other compounds in the insect intestines. Compared to the curves of the superworm and greater wax moth, yellow mealworm frass presented the lowest derivative weight, which indicated that the PS content in the yellow mealworm frass was lower and the degradation efficiency of PS was higher. Interestingly, yellow mealworm frass was detected at one obvious mass loss stage at 50–100 °C; however, it was absent in the other two larvae, which is in line with the findings of Yang et al. [12]. This was believed to be related to the material source that was used for the experiment.

The FTIR spectra were analyzed to study the oxidation and depolymerization of Styrofoam to frass in the gut of larvae. The results showed that PS-fed larvae’s frass presented more undulate peaks and obvious peaks at around 1075 cm^−1^, 1700 cm^−1^, and 3450 cm^−1^, thus representing C–O, C=O, and R–OH, respectively (Figure 2c). This finding suggested that the oxidation and depolymerization processes of PS occurred in the gut of the larvae [14]. Meanwhile, the frass of the three larvae produced similar FTIR spectra, except yellow mealworm at a 650 cm^−1^ peak, which represented weak ring-bending vibrations. These characteristic peaks indicated that the gut microbes of all three species’ larvae were able to degrade Styrofoam and that of yellow mealworm was found to destroy the benzene ring.

The ^1^H-NMR spectra for PS and frass revealed new peaks in the frass of larvae that fed on PS only. These peaks included δ 0, δ 0.9, δ 1.3, δ 5.4, and δ 7.2 and were detected in the region of hydrogen bong changes. In addition, the frass of yellow mealworm presented some unique peaks, such as δ 1.5–2.0, δ 3.5, and δ 6–7 (Figure 2d), which indicated that PS may be depolymerized strongly by the gut microbes of yellow mealworm. Meanwhile, this result was consistent with the FTIR conclusion that yellow mealworm could destroy the benzene ring.

### 3.3. Comparison of Gut Microbial Diversity

The gut microbiome of insects has an important role to play in their digestion process [29,30]. In order to determine and compare the changes of intestinal microbiome caused by feeding on polystyrene, the gut bacterial community richness, diversity, and composition of three species’ larvae were detected at three stages (0 d, 10 d, 20 d) using Illumina sequencing of 16S rRNA gene amplicons. A total of four-hundred and nine-thousand, six-hundred and sixty-four sequences of nine samples were obtained and listed in Table 2 with sampling coverage above 0.99, thus suggesting that Illumina sequencing was capable of detecting most of the reads. For greater wax moth and yellow mealworm, the OTU trends of the three stages showed a decrease in value, but the trend increased for superworm. The OTUs of the 0 d yellow mealworm gut bacterial community were relatively more than the other two groups. The Ace and Chao estimators were used to analyze the community richness. The results showed that Tmol_0 had the highest taxonomic richness, Gmel_0 and Gmel_10 had a lower taxonomic richness, while the other samples showed similar richness. Meanwhile, the Shannon and Simpson indexes of the Alpha diversity estimators were used to indicate community diversity. The results showed that there was a change in the gut microbe species diversity with respect to the prolongation of PS feeding time. Furthermore, the three species’ larvae showed different trends. The gut microbiome showed higher diversity in superworm and yellow mealworm before feeding them with PS. Upon PS feeding, a decrease in community diversity was observed in the case of superworm and yellow mealworm, but an increase was marked in the case of greater wax moth. The difference in diversity at 0 d might be due to their initial dietary differences; however, the difference on later days may be related to their gut ecophysiology [14]. Therefore, after feeding them the same food, i.e., polystyrene, the gut microbiome began to develop in a way that helped them digest it.

**Figure 3:**
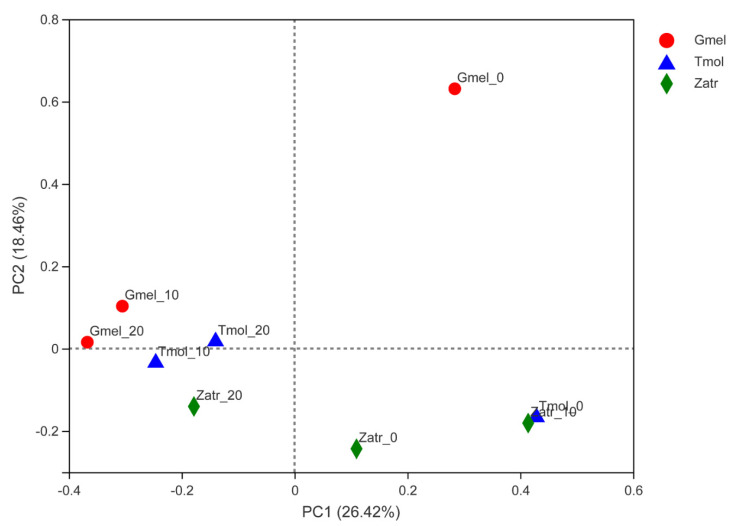
Principle co-ordinates analysis based on the Bray–Curtis distance among the nine groups of the three species’ larvae. Tmol: yellow mealworm, Gmel: greater wax moth, Zatr: superworm.

Beta analysis through principal co-ordinates analysis (PCoA) based on the Bray–Curtis distance was carried out to reveal the differences of the sample community composition among different groups of the three species’ larvae. According to Figure 3, feeding on PS had an obvious influence on the gut microbial composition. Gmel_0, Tmol_0, and Zatr_0 presented long-distance comparisons with 10 d and 20 d PS-fed groups of each species’ larvae, thus indicating a conspicuous difference before and after feeding PS. Meanwhile, Gmel_10 and Gmel_20 and Tmol_10 and Tmol_20 showed close distance comparisons with Gmel_0 and Tmol_0, which showed that the longer the PS feeding time was, the smaller the change in the gut microbial composition was for greater wax moth and yellow mealworm. The trend was also observed in *T. castaneum* larvae and *T. molitor* larvae before and after feeding with PS [14,17,28]. The distances of the Gmel_10, Gmel_20, Tmol_10, Tmol_20, and Zatr_20 community were found to be relatively close, which showed a similarity of their community composition. The findings showed that the degradation by the bacterial community that was involved in PS degradation in the intestines of the three species’ larvae was similar.

**Figure 4:**
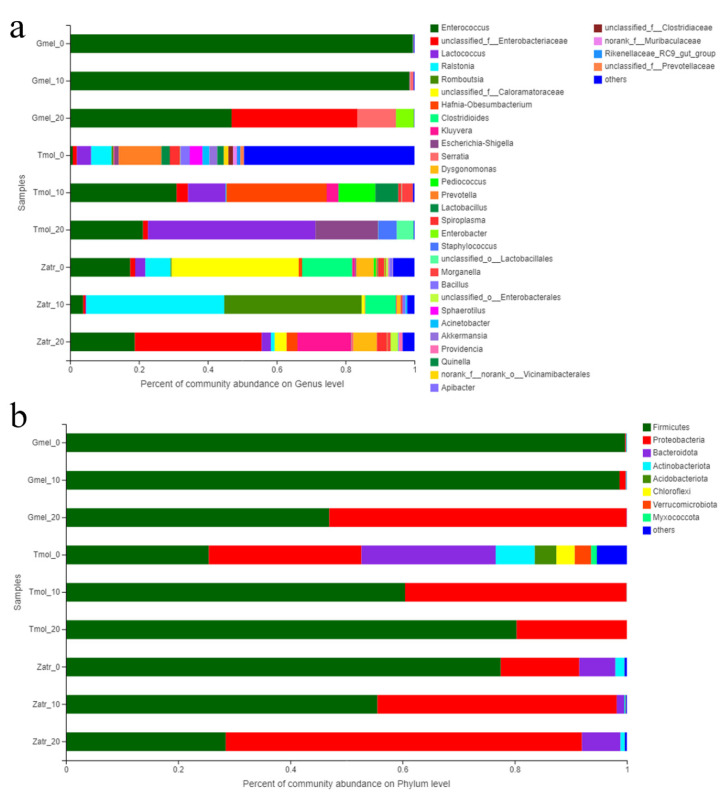
Community bar plot analysis of nine samples of the three species’ larvae based on genus (**a**) and phylum levels (**b**). Tmol: yellow mealworm, Gmel: greater wax moth, Zatr: superworm.

**Figure 5:**
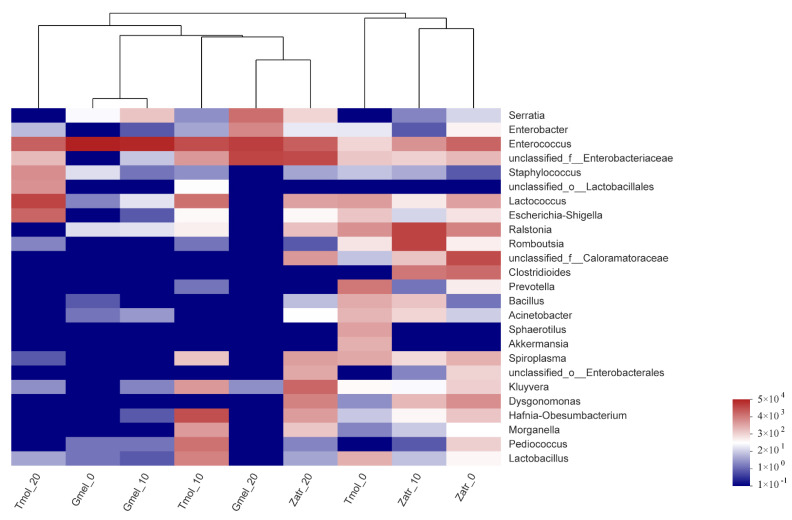
Gut bacterial community heatmap analysis of nine samples of the three species’ larvae. The analysis is on the basis of the top 25 abundant genera. The color intensity of the scale indicates the relative abundance. Tmol: yellow mealworm, Gmel: greater wax moth, Zatr: superworm.

The microbial community composition for the three species was analyzed by community bar plot analysis (Figure 4) based on the phylum and genus level and community heatmap analysis (Figure 5). The results showed that feeding on PS can induce the enrichment of some intestinal microorganisms, which may be the microorganisms responsible for PS digestion. For superworm, the core gut microbiome species were *Enterococcus*, *Enterobacteriaceae*, *Kluyvera*, and *Lactococcus*; for greater wax moth, they were *Enterococcus*, *Enterobacteriaceae*, *Serratia*, and *Enterobacter*; for yellow mealworm, they were *Enterococcus*, *Enterobacteriaceae*, *Escherichia-Shigella*, and *Lactococcus* (Figure 4a). The bacteria affiliated with Enterococcus and Enterobacteriaceae were found in all three species’ larvae of 20 d of PS feeding (Figure 4a and Figure 5), which indicated their crucial role in PS degradation. Figure 4b shows that *Firmicutes* and *Proteobacteria* were the predominant phyla in the guts of 20 d PS-fed groups. This result is consistent with the results of previous studies [16,28]. So far, many studies have successfully screened out the PS-degrading microbes from the insects’ guts. A similar study was carried out by Wang et al. [17], who investigated the gut microbiome of plastic- and bran-fed *T. castaneum* larvae and found that *Acinetobacter* sp. was strongly associated with PS degradation. Later, they were successful in isolating *Acinetobacter* sp. AnTc-1, which is a PS-degrading bacteria. Yang et al. [31] isolated a PS degrading bacterial strain *Exiguobacterium* sp. YT2 from the guts of *T. molitor* larvae. Brandon et al. [22] identified eight unique gut microorganisms associated with PS biodegradation including *Citrobacter freundii*, *Serratia marcescens*, and *Klebsiella aerogenes*. Woo et al. [32] isolated a PS-degrading strain *Serratia* sp. WSW from the gut flora of *P. davidis* larvae. These isolated strains belong to *Firmicutes* or *Proteobacteria*. Heatmap analysis also showed that four clusters were generated from the nine groups: Cluster I (Tmol_0, Zatr_0, and Zatr_10), Cluster II (Tmol_10, Gmel_20, and Zatr_20), Cluster III (Gmel_0 and Gmel_10), and Cluster IV (Tmol_20) (Figure 5), where each cluster implied a homology of microbial community structure. That is to say, the guts with a long PS feeding time tended to cluster together, while those with a short PS feeding time tended to cluster into another group. This result is consistent with the result of the Beta analysis.

### 3.4. Analysis of Degradation Products

In PS metabolism, the formation of intermediates represents the digestion of the PS by the larvae and its biodegradation [16]. Previous studies have reported that fatty acids and carboxylic esters represent the generated metabolic intermediates of plastics [33]. GC–MS analysis was conducted to determine the intermediates and products during PS metabolism in the gut and frass of larvae. The results revealed that a variety of acids and alcohols, such as 2-propenoic acid, benzenepropanoic acid, behenic alcohol, and phthalic acid, and long chain fatty acids, such as oleic acid, hexadecanoic acid, and octadecanoic acid (Table 3), were detected in the frass and gut of larvae fed on PS, thus representing the possible metabolism of the benzene structure and biodegradation of PS. In addition, 2-propenoic acid and behenic alcohol were detected in the frass of all three species’ larvae. Hexadecanoic acid was detected in the gut of all three species’ larvae. These results suggested that some of these gut microbes present in these three insect larvae were similar in their physiology and action of disrupting the bonding sites in the PS. Then, the microbes were found to be Enterococcus and Enterobacteriaceae, as mentioned above. Compounds with oxygen were detected in the PS samples, and some compounds appearing to be toxic and dangerous, such as phenol, heptane, and toluene, were also detected, which may be attributed to plasticizers, antioxidants, and other additives in the plastic foam [4]. However, these toxic and dangerous compounds were not detected in the gut and frass of larvae, suggesting that they may have been degraded by the larvae. Although the larvae degraded these compounds, which may also be harmful to them, this may also be a reason for the low survival rate of the treated group.

## 4. Conclusions

The eating and degradation of plastics by insect larvae is a new way to solve white pollution. The biodegradation of PS and the changes of the intestinal microbial diversity occurred in the insect guts, which may be closely related to these changed intestinal microorganisms. Currently, scientists have screened out the PS-degrading bacteria from the gut of some insect larvae. However, insect larvae and intestinal microorganisms have a mutually beneficial symbiotic relationship. After eating plastic, not only the intestinal microorganisms of insect larvae play an important role in the biodegradation of plastic, but also their enzyme system plays a crucial part in the degradation process. Therefore, further study is needed to determine the interaction between these insects and their gut microorganisms to reveal the degradation mechanism of plastic by insect eating.

## Figures and Tables

**Table 1:** Summary of PS biodegradation by the three species’ larvae.

Larvae	Initial Weight/Larva (g)	Feed	Weight Change at the End of the Test (%)	Survival Rate (%)	mg PS/Larva/d
Superworm	0.86 ± 0.021	PS	−51.67 ± 1.15	90.5 ± 0.5	2.78 ± 0.060 a
		Bran	+12.11 ± 3.37	93.67 ± 1.53	
Greater wax moth	0.21 ± 0.015	PS	−43.61 ± 4.67	27 ± 2.65	1.57 ± 0.066 b
		Beeswax	+18.89 ± 2.12	45.67 ± 3.06	
Yellow mealworm	0.08 ± 0.015	PS	−17.06 ± 5.28	75.5 ± 7.4	0.07 ± 0.009 c
		Bran	+25.92 ± 1.84	80.83 ± 7.11	

Values followed by different small letters (a–c) within a column are significantly different (*p* < 0.05).

**Table 2:** Quality of the samples and bacterial diversity analyses based on Illumina sequencing of the 16S rRNA gene amplicons.

Sample	Size	OTUs	Shannon	Simpson	Ace	Chao	Coverage
Gmel_0	52,347	35	0.180129	0.931893	50.41519	43.75	0.999543
Gmel_10	44,132	43	0.224694	0.925592	72.07289	60.1	0.999421
Gmel_20	48,381	13	1.336777	0.319691	13.375	13	0.99997
Tmol_0	36,796	1268	5.892504	0.009599	1269.646	1268.35	0.999756
Tmol_10	47,699	37	1.964937	0.197299	60.83233	42.6	0.999756
Tmol_20	43,830	27	1.605712	0.267592	38.36263	30.75	0.999817
Zatr_0	41,939	148	2.399686	0.189472	179.5835	168.6667	0.999024
Zatr_10	49,021	157	1.570013	0.32704	186.085	190.4762	0.998842
Zatr_20	45,519	161	2.337035	0.176703	199.1793	191.4412	0.998598

**Table 3:** Chemical compounds of PS and the frass, and gut of larvae analyzed by GC–MS.

Larvae	Sample	Chemical Compound
Greater wax moth	Frass	2-propenoic acid, behenic alcohol, benzenepropanoic acid, acetic acid, 6-tetradecanesulfonic acid
	Gut	Hexadecanoic acid, oxalic acid, phthalic acid, benzenepropanoic acid, benzoic acid
Yellow mealworm	Frass	2-propenoic acid, behenic alcohol, benzenepropanoic acid, silicic acid, [1,2,4]triazo[1,5-a]pyrimidine-6-carboxylic acid
	Gut	Hexadecanoic acid, 9-octadecenoic acid, z-8-methyl-9-tetradecenoic acid
Superworm	Frass	2-propenoic acid, behenic alcohol, 9-octadecenoic acid, phthalic acid, hexadecanoic acid, methoxyacetic acid
	Gut	hexadecanoic acid, stearic acid, phthalic acid, 6-octadecenoic acid
	PS	phenol, heptane, toluene, o-xylene, 3-(benzylthio)acrylic acid

## Data Availability

The data presented in this study are available upon request from the corresponding author.
